# Integrin-Ligand Interactions in Inflammation, Cancer, and Metabolic Disease: Insights Into the Multifaceted Roles of an Emerging Ligand Irisin

**DOI:** 10.3389/fcell.2020.588066

**Published:** 2020-10-26

**Authors:** Eun Jeong Park, Phyoe Kyawe Myint, Atsushi Ito, Michael G. Appiah, Samuel Darkwah, Eiji Kawamoto, Motomu Shimaoka

**Affiliations:** ^1^Department of Molecular Pathobiology and Cell Adhesion Biology, Mie University Graduate School of Medicine, Tsu, Japan; ^2^Department of Thoracic and Cardiovascular Surgery, Mie University Graduate School of Medicine, Tsu, Japan; ^3^Department of Emergency and Disaster Medicine, Mie University Graduate School of Medicine, Tsu, Japan

**Keywords:** integrin, ligand, irisin, inflammation, cancer, metabolic disease

## Abstract

Integrins are transmembrane proteins that mediate cellular adhesion and migration to neighboring cells or the extracellular matrix, which is essential for cells to undertake diverse physiological and pathological pathways. For integrin activation and ligand binding, bidirectional signaling across the cell membrane is needed. Integrins aberrantly activated under pathologic conditions facilitate cellular infiltration into tissues, thereby causing inflammatory or tumorigenic progressions. Thus, integrins have emerged to the forefront as promising targets for developing therapeutics to treat autoimmune and cancer diseases. In contrast, it remains a fact that integrin-ligand interactions are beneficial for improving the health status of different tissues. Among these ligands, irisin, a myokine produced mainly by skeletal muscles in an exercise-dependent manner, has been shown to bind to integrin αVβ5, alleviating symptoms under unfavorable conditions. These findings may provide insights into some of the underlying mechanisms by which exercise improves quality of life. This review will discuss the current understanding of integrin-ligand interactions in both health and disease. Likewise, we not only explain how diverse ligands play different roles in mediating cellular functions under both conditions via their interactions with integrins, but also specifically highlight the potential roles of the emerging ligand irisin in inflammation, cancer, and metabolic disease.

## Introduction (Integrin Biology)

**FIGURE 1:**
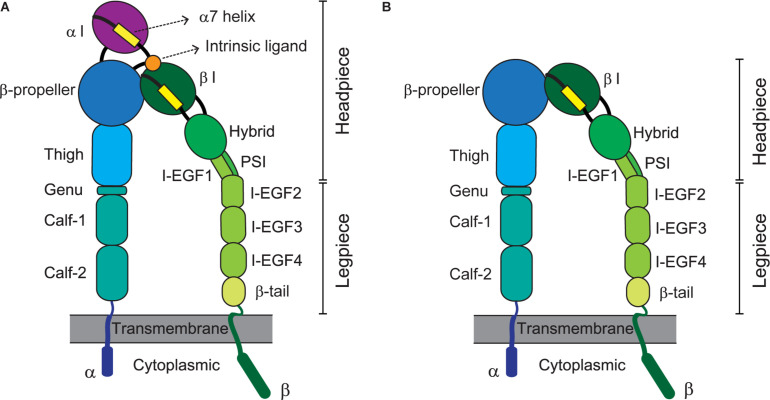
Integrin domains and structures. Illustrations depict the extended conformation of integrins bearing the headpieces with high-affinity domains. Representative structures of α I domain-containing integrins (e.g., αLβ2) and -deficient integrins (e.g., αVβ5) are shown at left **(A)** and right **(B)**, respectively. The integrin α/β heterodimer is composed of multiple functional domains in extracellular (headpiece plus legpiece), transmembrane, and cytoplasmic portions. I-EGF, integrin-epidermal growth factor; and PSI, plexin/semaphorin/integrin.

Integrins represent a large family of transmembrane cell-adhesion molecules that consist of non-covalently associated α/β heterodimers (Luo et al., 2007). Eighteen types of α chain and eight types of β chain associate with each other to form 24 different heterodimers (Takada et al., 2007). These can be classified into several groups including arginine-glycine-aspartate (RGD)-binding receptors, leukocyte-specific receptors, laminin receptors, and collagen receptors, depending on the traits of their ligands (Barczyk et al., 2010). By forging a molecular link between cells and their milieu [e.g., the extracellular matrix (ECM) or other cells], integrins advance cellular dynamic processes such as adhesion, migration, and extravasation (Geiger et al., 2001; Kechagia et al., 2019). Integrin-triggered intracellular signaling leads to cell division, survival, differentiation, and/or death, which are pivotal for vital phenomena (Meredith et al., 1993; Leone et al., 2005; Livshits et al., 2012). The activation of integrins before their adherence to cognate ligands, constitutes a variety of global, reversible, and cooperative conformational changes involving multiple structural domains (Shimaoka et al., 2002; Calderwood, 2004). An integrin’s ligand-binding affinity is enhanced in response to inside-out signals derived from activated G-protein or via coupling with other receptors (Takagi et al., 2002; Carman and Springer, 2003; Gahmberg et al., 2009; Springer and Dustin, 2012). Adhesion to ligands further stabilizes the high-affinity conformation of integrins for transmitting outside-in signaling, thereby achieving integrin clustering and strengthening adhesiveness (Takagi et al., 2002; Carman and Springer, 2003; Chen et al., 2006; Gahmberg et al., 2009). Specifically, their extracellular domains (e.g., the α-I domain for αLβ2 or the β-I domain for αVβ5) bind cognate ligands [e.g., intercellular adhesion molecule 1 (ICAM-1) for αLβ2 or fibronectin for αVβ5] to consequently signal bi-directionally across the cell membrane, which is called a hallmark of integrin activation (Takagi et al., 2002; Kim et al., 2003; Chen et al., 2006). Figure 1 depicts the structures and domains of two kinds of integrins, α I domain-containing αLβ2 (A) and α I-domain-lacking αVβ (B).

Integrin-mediated cell adhesion and migration are the integrated and controlled events required for physiologic and pathologic pathways (Gumbiner, 1996; Cox and Huttenlocher, 1998; Collins and Nelson, 2015; McMillen and Holley, 2015). In the case of leukocytes, during their migration to the tissue from bloodstream they undergo dynamic and sequential processes including tethering, rolling, firm adhesion, trans-endothelial migration (TEM), and extravascular migration (Ley et al., 2007). Molecularly, the selectins and integrins of leukocytes play roles in tethering and rolling on contact with the vascular endothelium. Thus, integrins are involved in such processes as slow rolling, firm adhesion, vascular crawling, TEM, and interstitial locomotion depending upon their activation states (Alon et al., 1995; Lawrence et al., 1995; Li et al., 1996; Chesnutt et al., 2006; Shulman et al., 2009; Walling and Kim, 2018).

The interaction of leukocyte integrins (e.g., αLβ2) with their cognate ligands (e.g., ICAM-1) expressed on endothelial cells is crucial to the entire process of homing to tissues (Ley et al., 2007). Leukocytes are recruited from the bloodstream to inflamed sites under pathologic conditions, an event that is also initiated by the interaction between selectins and their ligands, which leads to the aberrant activation of integrins (Zarbock et al., 2011). Understanding the structural and molecular mechanisms underlying integrin-mediated adhesion and migration has advanced the basic science required to apply this knowledge in clinical settings (Horwitz, 2012). Integrin-targeted therapeutics have mostly been designed to modulate integrin functions and thereby either suppress or promote cellular infiltration to the tissues. This has led to the development of effective inhibitory or agonistic drugs to counter the effects of pro- or anti-inflammations, respectively, although in some instances there have been side effects and/or problems of inefficacy (Lu et al., 2008; Baiula et al., 2019).

The majority of integrin heterodimers containing αV chain are known to correlate with cancer (Brooks et al., 1994; Weis and Cheresh, 2011). Specifically, the binding of αV integrins to the RGD motif within ECM proteins is a critical event for angiogenesis in cancer (Koistinen and Heino, 2002; Kaido et al., 2004; Pedchenko et al., 2004; Weis and Cheresh, 2011). Thus, RGD-binding αV integrins have garnered considerable attention as a target of cancer therapeutics. Below, we discuss in detail the implication of integrins and their ligands in inflammation, cancer, and metabolic disease.

## Integrin Ligands on the Cell Surface and in the ECM

Most integrins exhibit an ability to bind a wide range of ligands (Humphries et al., 2006). Located on the surfaces of cells are various sets of adhesion proteins involved in cell-cell interactions, as well as cell-ECM binding and interactions involving integrin receptors (Van Der Flier and Sonnenberg, 2001). These are collectively known as cell adhesion molecules (CAMs). CAMs function as ligands of integrins, facilitating the trafficking and homing of migratory cells such as leukocytes. Here, we briefly introduce several representative ligands for integrins.

**FIGURE 2:**
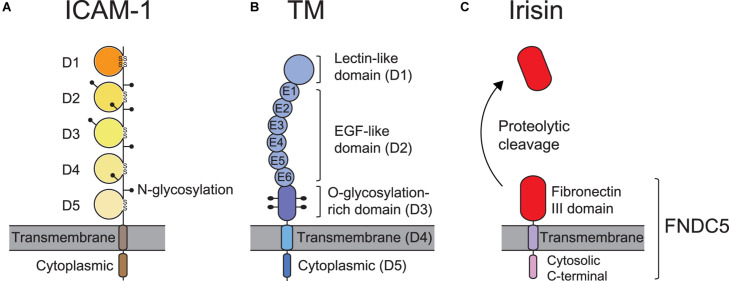
ICAM-1, TM, and FNDC5 domains and structures. **(A)** ICAM-1 consists of 5 immunoglobulin (Ig)-like domains (D1∼D5), a transmembrane domain, and a cytoplasmic domain and contains 8 N-linked glycosylation sites. The disulfide bonds in the Ig-like domains are formed between cysteine residues that stabilize the structure. **(B)** Thrombomodulin (TM) contains a lectin-like domain (D1), 6 epidermal growth factor (EGF)-like domains (D2), an O-glycosylation-rich domain (D3), a transmembrane domain (D4), and a cytoplasmic domain (D5). **(C)** Fibronectin type III domain-containing protein 5 (FNDC5) is composed of a fibronectin III domain (irisin), a transmembrane domain, and a cytosolic C-terminal domain. Irisin is produced by the proteolytic cleavage of FNDC5.

**FIGURE 3:**
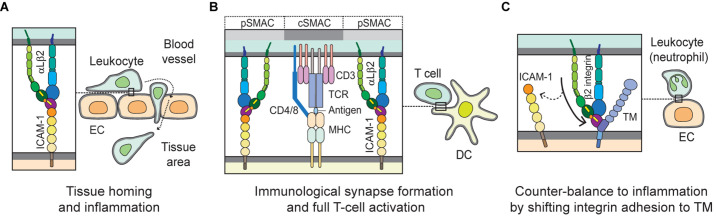
Biological interactions mediated by integrins with ICAM-1 and TM. **(A)** During leukocyte homing to normal or inflamed tissues, integrin αLβ2 plays a key role by interacting with its cognate ligand ICAM-1 on EC, in mediating slow rolling, firm adhesion and trans-endothelial migration, or extravascular movement. **(B)** When T cells migrate to the extravascular space in tissue, they probe cognate antigen-presenting DCs and subsequently form stable and mature immunological synapses. In the immunological synapse, the interaction of αLβ2 with ICAM-1 builds up a distinct marginal region called the pSMAC; TCR and auxiliary molecules are enriched in cSMAC, which may empower T cells to become fully activated. **(C)** The β2 integrin on leukocytes (e.g., neutrophils) binds to the O-glycosylation-rich domain (D3) of TM on EC. This interaction may help counter-balancing inflammation by shifting adhesion from ICAM-1 to TM. EC, endothelial cell; DC, dendritic cell; TCR, T-cell receptor; pSMAC, peripheral supramolecular activation cluster; and cSMAC, central supramolecular activation cluster.

ICAM-1 (CD54), the most biologically relevant member of the superfamily of immunoglobulin-like transmembrane glycoproteins, is a well-characterized molecule that has been implicated in pro-inflammatory immune responses (Muller, 2019). It is also known well as a receptor for rhinovirus in common colds, and as part of several receptors employed by *Plasmodium falciparum* in the infection of erythrocytes and vascular endothelium in Malaria (Berendt et al., 1989). ICAM-1 is natively expressed on endothelial cells, and its overexpression on endothelial, as well as antigen-presenting cells, is induced by surges of pro-inflammatory cytokines in several pathological states (Chirathaworn et al., 2002; Shaw et al., 2004). ICAM-1 on endothelial cells serves as a ligand for β2 integrins such as αLβ2 andαMβ2 expressed on leukocytes. Figure 2A illustrates the structure of ICAM-1. Interaction with ICAM-1 promotes the firm arrest and transmigration of leukocytes from the circulation into tissues (Muller, 2019; Figure 3A). The binding of αLβ2 on T cells to ICAM-1 on antigen-presenting cells, such as dendritic cells (DCs), forms the immune synapse that leads to full activation and polarization of T cells (Figure 3B; Wernimont et al., 2011; Morrison et al., 2015). Another member of β2 integrins, αDβ2, is expressed on macrophages, monocytes, neutrophils, eosinophils, basophils and a subset of lymphocytes. In addition, it is known to selectively bind to ICAM-3, though not to ICAM-1 (Van Der Vieren et al., 1995).

Vascular cell adhesion molecule 1 (VCAM-1; CD106) is expressed on activated endothelium and serves as a ligand for integrins, α4β1 (very late antigen-4; VLA-4) and α4β7. The activation of VCAM-1 is induced by factors such as pro-inflammatory cytokines (e.g., tumor necrosis factor-α; TNF-α), shear stress, high glucose concentrations and reactive oxygen species (ROS) (Cook-Mills et al., 2011). Initial encounters between the post-capillary endothelium and circulating leukocytes in the vascular bed are partly mediated by the binding of α4 integrins to membrane-bound VCAM-1 expressed on endothelium (Berlin et al., 1995). Such interactions not only aid rolling adhesion, but also extend the contact duration between leukocytes and the endothelium before extravasation. Integrin αDβ2 expressed on eosinophils has been shown to be a functional alternative binder to VCAM-1 involved in the static adhesion of eosinophils during chronic inflammation (Grayson et al., 1998). During leukocyte extravasation, the opening of tight junctions involves the signaling of VCAM-1 to VE-cadherin. Integrin α4β1 on the surface of leukocytes interacts with VCAM-1 and induces Rac-1 activation and the production of ROS, which subsequently leads to the phosphorylation of VE-cadherin by activated proline-rich tyrosine kinase (Pyk2). The result is a local loss of VE-cadherin function and the opening of junctions to facilitate TEM (Cain et al., 2010; Wessel et al., 2014). In relation to lymphocytes, the interaction of VCAM-1 on follicular dendritic cells and integrin α4β1 on B cells is necessary for their localization to lymphoid germinal centers and their subsequent differentiation (Freedman et al., 1990). Integrin α9β1 constitutively expressed in neutrophils binds to VCAM-1. The direct binding of α9β1 integrin to VCAM-1 has been implicated in the mechanism underlying survival and/or delayed neutrophil apoptosis and the maintenance of their physiologic function (Ross et al., 2006).

The recruitment of lymphocytes to the gut mucosa is mediated by the interaction between integrin α4β7 with mucosal addressin cell adhesion molecule 1 (MAdCAM-1) (Hamann et al., 1994). Expression of MAdCAM-1 is not only highly upregulated on inflamed venules in chronic inflammation such as colitis (McDonald et al., 1997), but also constitutively on the sinus-lining cells of the spleen, lactating mammary glands, post-capillary venules of the intestinal lamina propria, and the high endothelial venules of Peyer’s patches and mesenteric lymph nodes (Nakache et al., 1989; Kraal et al., 1995). In addition, the interaction between α4β1 and MAdCAM-1 plays a role in an alternative response to the recruitment of inflammatory T cells to the gut during chronic intestinal inflammation (Rivera-Nieves et al., 2005).

E-cadherin is a type-1 homophilic transmembrane protein that functions as a cell adhesion molecule on epithelial cells, serving an important function in the maintenance of epithelial integrity (Vleminckx and Kemler, 1999). E-cadherin acts as a ligand for integrin αEβ7 expressed on mucosal T cells. The binding of αEβ7 integrin to E-cadherin is believed to be vital to the retention of lymphocytes in mucosal epithelial regions (Schon et al., 1999). Studies have also demonstrated the heterotypic binding of non-leukocytic integrin, α2β1 and E-cadherin (Whittard et al., 2002).

Platelet endothelial cell adhesion molecule 1 (PECAM-1; CD31) expressed on platelets, endothelial cells, monocytes and neutrophils has been shown to bind to integrin αVβ3 in order to mediate the interaction of leukocytes with endothelial cells, which might be involved in angiogenesis (Piali et al., 1995). Recombinant human activated protein C (APC) was the first Food and Drug Administration–approved drug for the treatment of hyper-coagulation and excessive inflammation in severe cases of sepsis (Marti-Carvajal et al., 2012). This drug and further studies related to it were discontinued due to the excessive bleeding it induced in sepsis patients. However, the fact that APC regulates leukocyte migration and adhesion was the first major finding suggesting that coagulation factors are involved in regulating inflammation in vascular endothelial cells.

Endothelial cell protein C receptor (EPCR) was the first identified protein C receptor expressed on vascular endothelial cells (Fukudome and Esmon, 1994). When bound to an EPCR, this protein C changes to an APC with the help of the thrombin- thrombomodulin complex. APC plays an important role in coagulation homeostasis by inactivating the pro-coagulation factors Va and VIIIa (Bretschneider et al., 2007). In addition, soluble EPCR released from vascular endothelial cells binds to neutrophils through leukocyte-specific β2 integrin, contributing to anti-inflammatory effects (Kurosawa et al., 2000; Fink et al., 2013). Therefore, EPCRs on endothelial cells may be an important link between vascular inflammation and coagulation during sepsis. In fact, blood samples from patients with sepsis show elevated levels of soluble EPCR, indicating the pathological exacerbation of sepsis (Kurosawa et al., 1998). Increases in soluble EPCR may be involved in the homeostatic suppression of excessive inflammation during sepsis. Thus, soluble EPCR should be considered an option for sepsis treatment.

Thrombomodulin (TM), which is expressed largely on the luminal area of vascular endothelium, also possesses anti-coagulant and anti-inflammatory properties (Martin et al., 2013). It thus contributes to coagulation and inflammation crosstalk on vascular endothelial cells (Okamoto et al., 2012). Figure 2B shows TM domains and structures. The EGF-like domain (D2) in TM has an anticoagulant effect, while the lectin-like domain in TM has an anti-inflammatory effect. Leukocyte β2 integrins bind to the O-glycosylation-rich extracellular domain in TM (Figure 3C; Kawamoto et al., 2016); in fact, *in vivo* and *in vitro* experiments have shown that inhibiting adhesion between vascular endothelial cells and leukocytes produces an anti-inflammatory effect (Iba et al., 2013; Kawamoto et al., 2019). Because anticoagulant factors expressed on vascular endothelial cells function as integrin ligands, it is thought that they have a potential as novel drugs for the treatment of inflammation and hyper-coagulation in patients with sepsis.

Fibronectin (FN) is an abundant ECM protein containing three kinds of repeated modules and several binding sites that facilitate concurrent interactions with diverse extracellular components including integrins (Schwarzbauer and Desimone, 2011). Through the RGD motif, FN binds to integrins including α5β1 and αV-classes to participate in many biological functions (Johansson et al., 1997; Schwarzbauer and Desimone, 2011). αV-class integrins bound to FN mediate α5β1 binding to FN at different sites and α5β1 clustering, via eliciting intracellular signaling and mechano-sensing (Bharadwaj et al., 2017). Thus, while both integrins (αV and α5β1) compete for the binding of FN, ultimately they engage in cooperative crosstalk with each other to assemble cellular focal adhesion to ECM (Bharadwaj et al., 2017). Such integrin crosstalk is also mediated by an FN synergy site adjacent to the RGD motif (Benito-Jardon et al., 2017). In addition, lymphocyte interaction with FN via αV integrins (αVβ1/αVβ3) is pivotal for interstitial migration in dense tissues such as the skin, where they are upregulated under inflammatory conditions (Overstreet et al., 2013; Fernandes et al., 2020).

Collagen, another ample ECM component, functions as a coagulation factor. Collagen plays an important structural role in the extracellular matrix of many different tissues. It is also capable of interacting with the following integrins: α1β1, α2β1, α10β1, and α11β1 (Langholz et al., 1995; Zhang et al., 2003; Hamaia et al., 2017). Furthermore, collagen can efficiently attract platelets to damaged blood vessels to prevent extravascular bleeding. When blood vessels are damaged and the collagen fibers under vascular endothelial cells are exposed, α2β1 and αIIbβ3 integrins on the surfaces of platelets become activated. The α2β1 integrin binds to the collagen fibers and strengthens the adhesion of platelets to sites of damage (Morton et al., 1995). Additionally, αIIbβ3 integrin binds to fibrinogen, which contains an RGD sequence, in order to cross-link platelets. By this manner it promotes the formation of a platelet mass and modulates hemostatic functionality (Lefkovits et al., 1995).

Irisin, named after the Greek Goddess Iris, is an adipomyokine first discovered by Bostrom et al. (2012). It is an exercise-inducible peptide with 112 amino acids and a molecular weight of 12 kDa (Bostrom et al., 2012). It is a proteolytic-cleavage product of fibronectin type III domain-containing protein 5 (FNDC5), which is a glycosylated type I membrane protein containing a fibronectin III domain (Figure 2C; Ferrer-Martinez et al., 2002; Bostrom et al., 2012). The latter is regulated by the transcriptional regulator peroxisome proliferator-activated receptor-γ (PPAR-γ) co-activator 1α (PGC-1α) in skeletal muscles. In previous biochemical and X-ray crystallographic studies, irisin has been shown to be a homodimer structurally, with a beta sheet located between the monomers (Schumacher et al., 2013). Irisin, containing two sites for N-glycosylation at the Asn-7 and Asn-52 positions, can have a molecular weight of 22 or 25 kDa, depending on the addition of either one or two sugar chains (Zhang et al., 2014; Jedrychowski et al., 2015). Unlike other secreted molecules, both the structure and function of irisin are well preserved during the evolutionary process; for instance, mouse and human irisin are 100% identical (Bostrom et al., 2012; Aydin, 2014). Irisin is primarily secreted from skeletal muscles and adipose tissues. However, studies have shown that smaller quantities of irisin are produced from other organs such as the liver, pancreas, stomach, brain, heart, and spleen (Aydin et al., 2014; Martinez Munoz et al., 2018).

Many studies have suggested that serum irisin increases with physical activity, while the relationship between muscle FNDC5 (the precursor of irisin) mRNA and exercise remains debatable. The increment in serum irisin levels is accompanied by an increase in FNDC5 mRNA levels in skeletal muscles (Bostrom et al., 2012). A pilot study reported a 35% rise in plasma irisin levels in young healthy subjects, with the greatest increase following maximal workload (Daskalopoulou et al., 2014). Similarly, serum irisin levels rose after acute strenuous exercise (cycle ergometry) in both children and young adults (Loffler et al., 2015). In contrast, longer (6 weeks) or chronic (1 year) increases in physical activity did not affect irisin levels in school children (Loffler et al., 2015).

A randomized control trial revealed that a 3-times/week 26-week training program did not change pre-to-post training serum irisin levels (Hecksteden et al., 2013). Moreover, 8 weeks of endurance training by non-diabetic obese male subjects did not affect FNDC5 mRNA level in skeletal muscle (Besse-Patin et al., 2014). A cohort study demonstrated that only high-performance aerobic training caused an increase in FNDC5 mRNA in skeletal muscle (Lecker et al., 2012). These data heightened the speculation that serum irisin levels may be affected by the type of physical activity undertaken, the duration of training sessions, sample collection time and the time of sample analysis (Hecksteden et al., 2013; Tsuchiya et al., 2014; Loffler et al., 2015; Tsuchiya et al., 2015). A mass spectrometry analysis revealed that serum irisin levels averaged 3.6 ng/ml in sedentary young healthy adults, whereas they increased to 4.3 ng/ml in individuals who partook in aerobic training (Jedrychowski et al., 2015), though it varied with age, gender, and/or body mass index (BMI) (Al-Daghri et al., 2014; Yan et al., 2014; Loffler et al., 2015; Fukushima et al., 2016).

A growing number of studies on the association of irisin with different subunits of integrin have drawn attention to the possibility that irisin may act as a new ligand for the integrin. Differential hydrogen-deuterium exchange linked to mass spectrometry (HDX/MS) data revealed that irisin has a putative integrin-binding region at amino acids 60–76 and 101–108 (Kim et al., 2018). The motif proximal to that region (amino acid 55–57) showed a close structural similarity to the RGD motif in fibronectin, an established ligand for integrin αV (Kim et al., 2018). Although irisin lacked a key amino acid sequence (RGD), except for aspartic acid (Schumacher et al., 2013), blockage of the RGD motif by commercially available RGD-containing peptides showed a significant reduction of irisin-induced signaling pathways in bone cells (Kim et al., 2018). In addition, irisin treatment promoted the invasion and induced the extravillous differentiation of trophoblast cells by switching integrin α6 to integrin α1 (Drewlo et al., 2020). Irisin treatment in rats was shown to improve endometrial receptivity by inducing integrin αVβ3 (Li et al., 2019). However, the molecular mechanisms that underlie these effects and benefits are not well understood, partly due to the lack of knowledge regarding the irisin receptor.

Bostrom et al. (2012) hypothesized that irisin binds to as yet unidentified receptors. The irisin receptors were postulated to exist in the membrane of cardiomyoblasts (Xie et al., 2015), preadipocytes (Zhang et al., 2014), gastrointestinal cancers (Aydin et al., 2016), and pancreatic cancer cell lines (Liu et al., 2018). However, these studies were unable to identify the receptor upon which irisin acts and exerts its pleiotropic effects. Kim et al. (2018) demonstrated that treating osteocytes with doses of irisin as low as 10 pM induced the phosphorylation of focal adhesion kinase (FAK), the major intracellular molecule responsible for integrin signaling. Irisin treatment also increased the phosphorylation of zyxin, another downstream molecule of the integrin-signaling pathway, confirming that irisin acts on the integrin-signaling pathway (Kim et al., 2018). In fact, irisin was revealed to bind to several integrins in adipocytes and osteocytes, with integrin αV classes, including αVβ5 and αVβ1, exhibiting the strongest binding affinity (Kim et al., 2018). Treatment with cyclo RGDyK, a specific αVβ5 antagonist, abolished all irisin-induced signaling responses (Kim et al., 2018). This is the very first finding in which the irisin receptor was identified, and the authors suggested that αV integrin family members are probably the functional irisin receptors in other tissues as well (Kim et al., 2018).

Several studies have identified both the co-receptors and the integrins engaged in irisin binding. The integrins αVβ5 and αVβ1 were found to be involved in irisin-mediated FAK signaling in CD81^+^ adipocyte progenitor cells (Oguri et al., 2020). In this study, CD81 was found to form complexes with αVβ5 and αVβ1 and mediate irisin-induced FAK signaling (Oguri et al., 2020). Complete knockout or antibody-based blockage of either integrin β5 or β1 abolished the effect of irisin-induced FAK phosphorylation in adipocyte progenitor cells, suggesting that irisin plays a role as a ligand for αVβ5 and αVβ1 (Oguri et al., 2020). Moreover, irisin was proven to bind to the integrin αVβ5 receptor on gut epithelial cells both *in vitro* and *in vivo* (Bi et al., 2020a). Immunofluorescence analysis showed the co-localization of irisin and αVβ5, while the co-immunoprecipitation of both molecules revealed this integrin to be a receptor for irisin (Bi et al., 2020a). Treatment with cilengitide trifluoroacetate, an integrin αVβ5 inhibitor, reversed irisin’s protective effects against intestinal ischemia reperfusion (IR) injury both *in vitro* and *in vivo* (Bi et al., 2020a). Irisin-dependent restoration of gut barrier function following IR injury was shown to occur via the integrin αVβ5-AMPK-UCP2 pathway (Bi et al., 2020a). The same group demonstrated that irisin binding of αVβ5 exerted ameliorating effects on endothelial and microvascular damage, which was dependent on integrin-triggered signaling to AMPK-Cdc/Rac1 (Bi et al., 2020b). Furthermore, irisin binding to αVβ5 was shown to directly promote osteoclast generation and bone resorption (Estell et al., 2020). Thus, it has been proven that irisin is an authentic ligand for integrin αVβ5 (and/or αVβ1) and exerts beneficial effects on various tissues, presumably by interacting with this integrin. However, the possibility that irisin acts on other membrane receptors cannot be ruled out, and further research is needed to validate these findings.

## Integrins in Health and Disease I: Inflammation

As mentioned earlier, upon being activated integrins interact with their ligands and mediate rolling, adhesion, crawling, and transendothelial migration of leukocytes in order to undergo tissue homing and inflammation. Proper regulation of integrin function is essential for controlling inflammatory responses (Herter and Zarbock, 2013). In order to home to a site of inflammation, leukocytes roll on the endothelium of blood vessels via interactions with selectins and chemokines on the endothelial surface. These interactions trigger inside-out and outside-in signaling cascades that result in the activation of integrins on the leukocytes into high-affinity conformations that facilitate the binding of integrins to their coordinate ligands (e.g., αLβ2/ICAM-1; α4β1/VCAM-1) and firm adhesion of leukocytes to the endothelial wall (Campbell et al., 1998; Herter and Zarbock, 2013). Thereafter, leukocytes move slowly along the surface of the endothelium in a process termed crawling, which ensures the location of an appropriate extravasation site for said leukocytes (Schenkel et al., 2004; Phillipson et al., 2006). Crawling is predominantly mediated by the integrin αLβ2 or αMβ2 depending on the leukocyte subtype (Sumagin et al., 2010). Subsequently, integrins αLβ2, αMβ2, and α3β1 interact with various ligands, including ICAM-1/2 and junctional adhesion molecules, to facilitate transendothelial migration and detachment of leukocytes (Williams et al., 2011; Subramanian et al., 2016). Figure 3A depicts the interaction of integrin (e.g., αLβ2) and ligand (e.g., ICAM-1) during leukocyte homing to normal or inflamed tissues.

Integrins play a significant role in adaptive immunity. As aforementioned, successful antigen presentation and T-cell activation by antigen-presenting cells at the specialized cell-cell interface known as the immunological synapse require firm adhesion of the cells (Makgoba et al., 1988), as well as co-stimulation in addition to the classical interactions between the T cell receptor (TCR) and the peptide-MHC complex (Van Seventer et al., 1990). The binding of integrin αLβ2 to ICAM-1 not only provides sustained contact between the T cells and antigen-presenting cells, but also induces the outside-in signaling cascade needed for full T-cell activation, proliferation, and differentiation (Makgoba et al., 1988; Van Seventer et al., 1990; Morgan et al., 2001). Figure 3B illustrates the immunological synapse formed by the αLβ2-ICAM-1 interaction required for full T-cell activation.

Resolution of an acute inflammatory response is pivotal to maintaining tissue homeostasis and serves to prevent the onset of an aberrant chronic inflammatory state. This is achieved largely through the removal of apoptotic cells (mainly neutrophils) by phagocytes in a process termed efferocytosis, in which integrins have been shown to be key player (Greenlee-Wacker, 2016). Additionally, the clearance of apoptotic immune cells re-programs phagocytic cells to a pro-resolving phenotype (Ortega-Gomez et al., 2013).

The expression of integrins αVβ3 and αVβ5 on myeloid cells (macrophages and dendritic cells, respectively) boosts the clearance of apoptotic cells, whereas αVβ8 induces the expansion of regulatory T (Treg) cells in a transforming growth factor β1 (TGF-β1)-dependent manner (Albert et al., 1998; Hanayama et al., 2002; Paidassi et al., 2011). This occurs in tandem with the loss of αV, thereby triggering the development of inflammatory bowel disease (IBD) and autoimmunity (Lacy-Hulbert et al., 2007; Travis et al., 2007). In a lipopolysaccharide (LPS)-induced lung inflammation model, intra-tracheal instillation of apoptotic cells was shown to elicit phagocytosis of these cells by CD11c^+^CD103^+^ DCs, which prime the expansion of Treg cells and mitigate lung inflammation (Zhang et al., 2020). Loss of integrin αV by these DCs results in impaired phagocytosis of apoptotic cells, suppression of TGF-β1 production and Treg-cell expansion, and consequently exacerbation of lung inflammation. Although αV integrin has been reported to be a major player in the onset of fibrosis in several organs (Conroy et al., 2016), it has emerged that fibroblast-specific loss of αV integrin may modulate localized inflammatory responses. In fact, it has been demonstrated that fibroblast-specific deletion of αV integrin decreases type 17-driven (but not type-2 driven) liver and lung fibroses. These fibrosis models further revealed not only a concurrent increase in type 2 inflammation markers such as interleukin-13 (IL-13), but also an accumulation of eosinophils in the lungs and livers of mice lacking αV on their fibroblasts. This suggests that blockade of αV integrin in the quest to ameliorate fibrosis may trigger pathologic type 2 inflammatory responses (Sciurba et al., 2019).

ECM stiffening and perpetual sedimentation are characteristics of fibrosis, which involves the stimulation of integrin signaling and dysregulation of matrix metalloprotease (MMP)-mediated ECM degradation (Bonnans et al., 2014). Under conditions of chronic inflammation, interstitial cytokines such as TGF-β1 and IL-13 induce fibroblasts to upregulate ECM production, resulting in fibrotic progression of the inflamed tissues (Biancheri et al., 2014; Bonnans et al., 2014). Thus, ECM remodeling and deposition are critical to building up the integrated process of fibrosis (Herrera et al., 2018).

Integrin β1 has also been shown to modulate lung inflammation, since deletion of β1 from type 2 alveolar epithelial cells (AECs) causes emphysema and a surge of macrophages in the lungs of adult mice. Furthermore, in younger mice, β1-loss results in defects in tight junctions and decreases in claudin-3 and claudin-4 in type 2 AECs. In addition to increased proliferation of type 2 AECs, a feature of lung injury, β1-deficient type 2 AECs showed an increased release of NF-κB-dependent cytokines and other inflammatory mediators, including those involved in macrophage chemotaxis both *in vivo* and in *ex vivo* cultures (Plosa et al., 2020). Blockade of integrin α4β7 has shown therapeutic benefits in IBD (Feagan et al., 2013; Sandborn et al., 2013). However, 36–54% of patients were refractory to this treatment (Peyrin-Biroulet et al., 2019). Sun et al. (2020) has reported that deficiency of integrin β7 in IL-10 null IBD mice exacerbates both spontaneous and induced colitis by inhibiting the homing of Treg cells to the gut and its associated lymphoid tissues. This suggests that although therapeutic blockade of β7 integrin limits the homing of conventional T cells to the gut, it may in turn abrogate the recruitment of Treg cells to the gut where they ameliorate inflammation.

Dysregulation of integrin αVβ3 and the integrin-associated glycoprotein CD47 have been shown to contribute to the development of osteoarthritis. Transcriptomic and proteomic analyses of osteoarthritic joint tissues from both humans and mice showed elevated levels of integrin αVβ3, as well as CD47 and multiple ligands of αVβ3 including cartilage oligomeric matrix protein, fibronectin, and vitronectin. Genetic deletion or pharmacological blockade of αVβ3 and CD47 and other downstream targets of integrin activation, such as FAK, protected mice against synovitis and cartilage degradation. For example, macrophages from integrin and CD47-deficient mice expressed lower levels of inflammatory and degradative mediators (Wang et al., 2019). Similarly, in a mouse sepsis model, deficiency in integrin β3 was shown to mitigate lung, kidney, and liver damage and enhance survival (Chen et al., 2016, 2020). *In vitro* treatment of β3-null, β3 neutralizing antibody or inhibitor pre-exposed peritoneal macrophages with LPS significantly reduced the secretion of TNF-α and IL-6, suggesting that integrin β3 may regulate the production of cytokines by these innate immune cells. Mechanistically, β3 was shown to upregulate CD14, a key factor that enhances toll-like receptor 4 (TLR4)-LPS interactions during the inflammatory response to sepsis. Thus, β3 may serve as a putative therapeutic target in ameliorating sepsis (Chen et al., 2020). In an ovalbumin-induced asthma murine model, administration of recombinant milk fat globule epidermal growth factor 8 (MFG-E8) suppressed both lung inflammation and airway remodeling by binding with its receptor integrin β3 (Zhi et al., 2018). It has been shown that integrin αMβ2 is essential for the adherence of neutrophils to endothelial cells as well as for transmigration (Hahm et al., 2013). In models of sterile vascular and hepatic inflammations, neutrophil myeloperoxidase was shown to downregulate the expression of integrin αMβ2 on activated neutrophils and, moreover, negatively regulate the transmigration of neutrophils as well as their interaction with inflamed endothelium (Tseng et al., 2018).

## Integrins in Health and Disease II: Cancers

Integrins are involved in almost every step of cancer progression, including cancer initiation and proliferation, local invasion and intravasation into the vasculature, survival of circulating tumor cells, priming of the metastatic niche, extravasation into secondary sites and metastatic colonization of new tissues (Hamidi and Ivaska, 2018). Until now, a vast literature has documented altered integrin expression in different cancer types. Indeed, the expression of α3β1, α4β1, α5β1, α6β4, αVβ3, αVβ5, αVβ6, and αVβ8 are thought to correlate with metastasis and poor patient prognosis (Nieberler et al., 2017; Hamidi and Ivaska, 2018). Although the integrin regulation between cancer cells and the microenvironment is quite complicated, some key contributions to cancer progression, in particular to metastasis, have been established. Here, we will discuss selected known and emerging roles of integrins, and their relevance, to events critical for cancer progression and metastasis.

Epithelial-mesenchymal transition (EMT) is the first step of cancer invasion and metastasis. EMT involves the loss of epithelial characteristics via the down-regulation of proteins such as E-cadherin and a shift toward a fibroblast-like phenotype via the expression of mesenchymal proteins such as α-smooth muscle protein (α-SMA), MMPs, and enhanced motility (Brabletz et al., 2018). In this context, integrins play an important role in the induction of EMT and in mediating some of its effects. One of the major EMT inducers is TGF-β, which is a cytokine capable of exerting immunosuppressive, anti-inflammatory, and pro-fibrotic activities. TGF-β is produced and secreted to the extracellular space as an inactivate precursor in which mature TGF-β is caged in latency-associated protein (LAP), thereby confining its bioactivities (Travis and Sheppard, 2014). LAP contains an integrin-binding RGD motif, which allows αV integrins to bind and impose a mechanical force to open the LAP cage (Travis and Sheppard, 2014). Upon binding to the receptor, mature TGF-β induces the downregulation of epithelial proteins, such as E-cadherin, and the upregulation of mesenchymal proteins such as N-cadherin. In addition, integrin α3β1 expression is required for TGF-β-stimulated small mothers against decapentaplegic (SMAD) signaling, which leads to EMT (Kim et al., 2009; Alday-Parejo et al., 2019). On the other hand, epithelial cell stimulation with TGF-β leads to a down-regulation of β4 integrin, which is a typical epithelial integrin essential for epithelial integrity and stability, thus facilitating migration (Yang et al., 2009; Alday-Parejo et al., 2019). Cancer-cell invasion occurs preferentially along pre-existing ECM tracks followed by tissue remodeling.

Collagen crosslinks and ECM solidification strengthen β1-integrin clustering and signaling and focal adhesions, which comprise the dynamic and integrated processes that are prerequisite for breast cancer cell invasion (Levental et al., 2009). In addition, laminin chains increased by tumor-associated fibroblasts provide the altered ECM deposition favorable for α6β4-mediated migration of cervical cancer cells (Fullar et al., 2015). This study demonstrates the roles played by interstitial ECM remodeling and the cross-talk that occurs between tumor and stromal cells in invasive cancers. Therefore, ECM remodeling and degradation, in concert with integrin function, are critical for invasion. MMPs are the proteolytic enzyme of collagen, fibronectin, and laminin—the main components of ECM. In a cervical cancer-cell line, integrin α5β1-fibronectin interaction was found to induce the expression and activation of pro-MMP9 and moderate changes to pro-MMP2 activity involving the FAK, integrin-linked protein kinase, ERK, phosphoinositide 3-kinase (PI3K), and NF-κB signaling cascades (Ganguly et al., 2013). Interaction of integrin α5β1 with laminin also induced MMP-9 expression and activated the signaling cascade (Ganguly et al., 2013). This ligand-integrin interaction was also found to accelerate cell migration. In addition, not only integrin α5β1 but also integrin αVβ3 has been linked to MMP activity. For instance, integrin αVβ3 expression is a major determinant of breast cancer cell bone metastasis (Takayama et al., 2005; Ganguly et al., 2013). Moreover, integrin αVβ3 is found as a modulator of MMP-2 activation and lymph-node metastasis (Hosotani et al., 2002; Ganguly et al., 2013).

Angiogenesis is another important factor in cancer progression and metastasis. In general, tumors lacking blood circulation grow to 1–2 mm^3^ in diameter and then stop; however, when placed in an area where angiogenesis has arisen they can grow > 2 mm^3^ (Folkman, 1986). In the absence of vascular support, tumors may become necrotic or even apoptotic due to insufficient supplies of oxygen and nutrition. Furthermore, angiogenesis not only provides nutrients for the tumor to grow, but also supports an escape route for tumor cells to enter the circulation. The mechanism underlying angiogenesis is mostly modulated by chemical stimuli such as vascular endothelial growth factor (VEGF), fibroblast growth factor (FGF), angiopoietins, epidermal growth factor (EGF), etc., all of which are plentiful at tumor sites (Teleanu et al., 2019). In particular, VEGF and FGF and their receptors are the most potent activators of angiogenesis. In this context, three endothelial integrins play crucial roles: αVβ3, αVβ5, and α5β1. VEGF-mediated angiogenesis occurs via αVβ5, while FGF-mediated angiogenesis occurs via αVβ3 and α5β1 (Foubert and Varner, 2012; Bianconi et al., 2016). In preclinical studies, the inhibition of angiogenesis with αVβ3 antagonists suppressed tumor progression, raising high expectations that an αVβ3 blockade may represent a valuable anti-cancer strategy (Liu et al., 2008). However, while integrin αVβ3-deficient mice show normal developmental angiogenesis, there is an increase in pathological angiogenesis. One hypothesis to explain this disparity is that animals lacking integrin αV develop compensatory pathways for VEGF signaling to permit the onset of angiogenesis during embryogenesis (Foubert and Varner, 2012). In clinical settings, administration of αVβ3 inhibitor “cilengitide” was unable to exert a potent therapeutic effect due to the induction of enhanced angiogenesis in the tumor environment (Bianconi et al., 2016; Wick et al., 2016). These reports demonstrate the functional complexity of integrins in regulating angiogenesis.

In addition to EMT and angiogenesis, a specialized microenvironment called the metastatic niche is important for disseminated cancer cells to adapt and survive in new tissues. Metastatic niches can be induced by primary tumors even before disseminated cancer cells reach the peripheral tissues to promote their survival and outgrowth. This implies some cross-talk between the primary tumor and peripheral tissues. In regards to the aforementioned mechanism, exosomes released by cancer cells play a vital role in cancer metastasis, contributing to the formation of metastatic niches, influencing cancer cells and the microenvironment, and determining specific organotropic metastasis (Hoshino et al., 2015). Breast cancer exosomes expressing high levels of αVβ5 were shown to disseminate to liver tissue containing a fibronectin-enriched ECM, whereas those expressing high levels of α6β4 disseminated to lung tissues containing laminin-enriched ECM (Hoshino et al., 2015; Shimaoka et al., 2019; Myint et al., 2020). The proposed mechanism underlying pre-metastatic niche formation involves the activation of the pro-inflammatory S100 genes found in lung and liver tissues (Hoshino et al., 2015). While exosomal transfer of the intact integrin-signaling complex has yet to be demonstrated, exosomally transferred integrin proteins could trigger the signals that lead to S100 activation by using Src Kinases derived from either exosomes or target cells (Hoshino et al., 2015). In this way, cancer-associated exosomes establish the organotropic pre-metastatic niche. These findings suggest that the interruption of integrin functions and ligand-dependent signaling could be a promising approach for developing therapeutics to combat various cancers.

## Irisin Biogenesis and Biological Aspects

**FIGURE 4:**
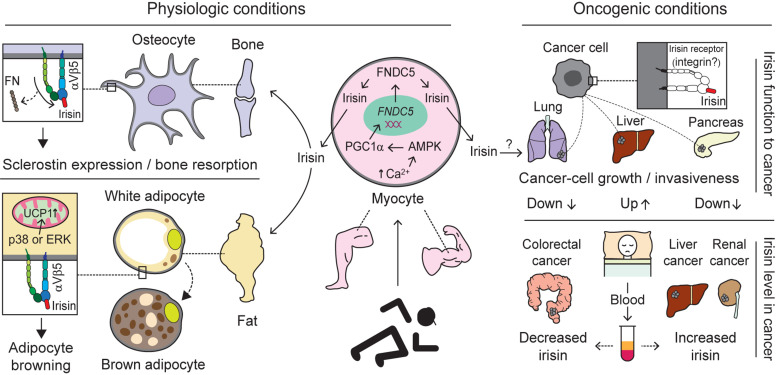
Biological interactions mediated by integrins with irisin. Exercise-induced production and release of irisin from the myocytes of skeletal muscle through activation of the AMPK-PGC1α-FNDC5 axis alters biological pathways via interaction with integrin (Bostrom et al., 2012; Shan et al., 2013; Gamas et al., 2015). Under physiologic conditions, irisin migrated to the bone via blood circulation preferentially, compared to FN, interacts with integrin αVβ5 acting on osteocytes to induce sclerostin expression and bone resorption. Moreover, irisin binding to Vβ5 on adipocytes is believed to facilitate adipocyte browning through the activation of p38 or ERK followed by increases in UCP1 (Zhang et al., 2014; Kim et al., 2018). Under oncogenic conditions, irisin binding to cancer cells is thought to have different impacts, although irisin receptors specific to cancer cells exist remains an open question. Specifically, irisin plays a role in inhibiting the growth or invasiveness of lung- and pancreatic-cancer cells (Shao et al., 2017; Liu et al., 2018), whereas it stimulates liver-cancer cells to promote tumor progression (Shi et al., 2017). In addition, the levels of irisin in blood (serum or plasma) of cancer patients appear to vary depending on which organ is primarily cancerous. For example, increased levels of irisin were found in liver- and renal-cancer patients (Gaggini et al., 2017; Altay et al., 2018), while decreased levels were detected in colorectal-cancer patients (Zhu et al., 2018). This suggests irisin levels may serve as a diagnostic marker for different cancers. AMPK, adenosine monophosphate (AMP)-activated protein kinase; PGC1α, peroxisome proliferator-activated receptor coactivator 1α; FNDC5, fibronectin type III domain-containing protein 5; FN, fibronectin; ERK, extracellular signal-regulated kinase; and UCP1, uncoupling protein 1.

A collective number of molecules seems to co-operate in the synthesis and secretion of irisin, a cleaved fragment of FNDC5 protein, via the AMPK-PGC-1α-FNDC5 axis (Bostrom et al., 2012; Shan et al., 2013; Gamas et al., 2015). Skeletal muscle contractions due to exercise increase levels of cytoplasmic calcium, which in turn activates AMP-activated protein kinase (AMPK). Moreover, the rise in the AMP/ATP ratio also phosphorylates AMPK (Mu et al., 2001; Jessen and Goodyear, 2005), which subsequently upregulates PGC-1α. The latter is known to interact with and coactivate several transcription factors and nuclear receptors, among which FNDC5 is one of the downstream molecules (Lira et al., 2010; Bostrom et al., 2012; Gholamnezhad et al., 2020). Consequently, FNDC5 expression is upregulated (Bostrom et al., 2012). Moreover, muscle contractions initiate the cleavage of FNDC5 with the help of an unknown protease (Figure 4). Although the cleavage and release of irisin is believed to occur in a manner similar to those of transmembrane polypeptides, such as EGF and TGF-α (Bostrom et al., 2012), the molecular mechanism underlying proteolytic cleavage and the enzyme(s) involved remains unknown. After proteolytic cleavage, the C-terminal tail of FNDC5 is anchored in the cytoplasm, whereas the extracellular N-terminal part is released into the circulation as irisin (Ferrer-Martinez et al., 2002; Teufel et al., 2002; Bostrom et al., 2012; Wrann et al., 2013).

Irisin has been shown to exert its pleiotropic effects on different tissues and signaling pathways. Many studies have linked irisin’s diverse effects with the AMPK pathway. In fact, via this pathway irisin has been shown to decrease inflammation and insulin resistance (Xiong et al., 2018), induce the browning of fat (Shan et al., 2013), lower blood pressure (Fu et al., 2016), promote differentiation and improve trophoblast functions in human placenta (Drewlo et al., 2020). Irisin treatment increased the expression of sclerostin in osteocytes to induce bone resorption (Kim et al., 2018). Irisin induces the browning of fat via the upregulation of UCP1 mRNA in subcutaneous adipose tissue (Kim et al., 2018). In addition, it reduces obesity and improves glucose tolerance in mice fed a high-fat diet (HFD) (Bostrom et al., 2012). Irisin’s browning effect is exerted via p38 and ERK signaling (Zhang et al., 2014; Figure 4). Treatment with recombinant irisin significantly increases phosphorylated p38 and phosphorylated ERK in both primary rat and 3T3-L1 adipocytes, while cotreatment with an inhibitor of either p38 or ERK or both abolishes the irisin-induced upregulation of UCP1 expression (Zhang et al., 2014). Irisin has been shown to promote Nkx2.5-positive cardiac progenitor cell-induced cardiac regeneration, neovascularization and functional improvements in the ischemic heart (Zhao et al., 2019). Moreover, administration of irisin promotes the proliferation of human umbilical vein endothelial cells and cord formation via the ERK pathway (Wu et al., 2015), suggesting that irisin plays a role in guarding against cardiovascular diseases.

In the context of the brain, FNDC5/irisin is expressed in the hippocampus, cortex and cerebrospinal fluid of wild-type C57BL/6 mice (Lourenco et al., 2019). An Alzheimer’s disease (AD) model of mice revealed a lower level of irisin in the brain, while conditional knockout of brain irisin resulted in impaired long-term potentiation and novel object recognition memory in mice. Boosting of brain irisin by intracerebroventricular infusion rescues memory impairment and provides protection against synaptic plasticity in AD mice (Lourenco et al., 2019). Another group showed that irisin binds with amyloid precursor protein at the N-terminal and that overexpression of FNDC5 in cells significantly decreased the secretion of amyloid-β protein into the media *in vitro* (Noda et al., 2018). In addition, pharmacological, but not physiological, concentrations of irisin (50–100 nM) increase the proliferation of a mouse hippocampal cell line, H19-7 HN, without influencing markers of neurite outgrowth or synaptogenesis *in vitro*. The proliferation of H19-7 HN cells occurs through the activation of the signal transducer and activator of transcription 3 (STAT3), but not the AMPK and/or ERK, signaling pathway (Moon et al., 2013).

## Roles of Irisin in Inflammation, Cancer, and Metabolic Disease

Irisin has been shown to exhibit anti-inflammatory properties in adipocytes and in immune cells. A cohort study showed that irisin levels were negatively associated with TNF-α levels in adipocytes (Moreno-Navarrete et al., 2013). Irisin has been shown to stimulate the proliferation and phagocytic activity of macrophages, while decreasing ROS overproduction in macrophages. Irisin treatment inhibited LPS-induced M1 macrophage polarization and inflammatory cytokine production both in RAW264.7 cells and in peritoneal macrophages (Xiong et al., 2018). Overexpression of FNDC revealed the attenuation of inflammatory cytokines in HFD-induced obese mice (Xiong et al., 2018). Another study also illustrated how pre-treatment with irisin lowered LPS-induced cytokine production in RAW264.7 cells via the downregulation of TLR4 and myeloid differentiation primary response protein (MyD88) levels, which consequently lowered the phosphorylation and/or activation of molecules involved in downstream signaling pathways (Mazur-Bialy et al., 2017). These data suggest that irisin plays an important role in both physiological and pathological conditions.

Concerning cancers, many studies have suggested that exogenous irisin exerts anti-tumor and anti-metastatic effects (Kong et al., 2017; Shao et al., 2017; Liu et al., 2018), while others have reported its stimulatory effects on tumorigenesis (Shi et al., 2017). Irisin has been shown to suppress the progression and invasion of glioblastoma by inducing p21 and tissue factor pathway inhibitor-2 (Huang et al., 2020). In breast cancer subjects, serum irisin levels were significantly lower compared with healthy volunteers (Provatopoulou et al., 2015). The authors estimated that a 1 unit increase in irisin levels could reduce the probability of breast cancer by almost 90% (Provatopoulou et al., 2015). Moreover, serum irisin was shown to play a protective role against spinal metastasis in breast cancer patients after adjusting for age and BMI (Zhang et al., 2018). Those breast cancer patients without spinal metastasis had significantly higher serum irisin levels compared to those with spinal metastasis (Zhang et al., 2018). However the levels of irisin in the blood (serum or plasma) of cancer patients appear to vary depending on which organ is primarily cancerous. For example, increased levels of irisin were found in liver- and renal-cancer patients (Gaggini et al., 2017; Altay et al., 2018), while decreased levels were detected in colorectal- and breast-cancer patients (Provatopoulou et al., 2015; Zhang et al., 2018; Zhu et al., 2018; Figure 4). This suggests irisin levels may serve as a diagnostic marker for different cancers.

It is well-known that the PI3K/AKT pathway is elevated in many cancers and is responsible for cancer cell growth, proliferation and survival (Vivanco and Sawyers, 2002). Irisin was found to inhibit the proliferation, migration and invasion of lung cancer cells by reversing the EMT via PI3K/AKT pathway activation (Shao et al., 2017). In line with that, the anti-metastatic effects of irisin was demonstrated on osteosarcoma cells. Irisin treatment reversed IL-6 induced EMT in U2OS and MG-63 osteosarcoma cells, and inhibited the migration and invasion of cancer cells through the STAT3/Snail-signaling pathway (Kong et al., 2017). Non-modified irisin decreased cancer cell counts, viability and migration through upregulated caspase-3/7 activity and NF-*K*B suppression in malignant breast cancer cell lines (Gannon et al., 2015). In this study, irisin was shown to enhance doxorubicin activity in malignant breast cancer cells, but not in non-malignant breast cancer cells (Gannon et al., 2015). In addition, irisin has been shown to suppress pancreatic cancer cell growth and proliferation (Liu et al., 2018), and to enhance doxorubicin-induced pancreatic cancer cell apoptosis through the upregulation of caspase-3 expression and the reduction of anti-apoptotic B-cell lymphoma 2 (Bcl-2) and Bcl-xL expression (Liu et al., 2019). Irisin potentiated the effects of chemo-therapeutic agents in pancreatic cancer cells via suppression of the PI3K/AKT/NF-*K*B signaling pathway (Liu et al., 2019; Figure 4).

In contrast, use of irisin in obesity-related cancer lines such as colon, esophageal, endometrial and thyroid cancer cells showed no effects in term of cell proliferation or invasion (Moon and Mantzoros, 2014). Irisin stimulated the proliferation and invasion of human hepatocellular carcinoma cells (Shi et al., 2017). There was an increase in irisin level in cancerous liver tissues obtained from hepatocellular carcinoma (HCC) patients, although there was no difference in serum irisin level between HCC patients and healthy volunteers (Shi et al., 2017). Moreover, irisin was found to not only increase proliferation and invasion of HepG2 cells, but also to decrease doxorubicin-induced HepG2 cell apoptosis, effects that occurred via the PI3K/AKT pathway (Shi et al., 2017; Figure 4). Although both the anti- and pro-tumor effects of irisin seem to involve the PI3K/AKT pathway, the underlying mechanism that might explain these discrepancies remains unknown (Moon and Mantzoros, 2014; Gannon et al., 2015; Shao et al., 2017; Shi et al., 2017; Liu et al., 2019). Further studies are required to validate the differential effects of irisin on different cancers.

Since the discovery that irisin might exert potentially beneficial effects on metabolic diseases (Bostrom et al., 2012), many researchers have tried to determine the nature of the association between serum irisin levels and these diseases. Obese subjects had significantly higher serum irisin levels compared to non-obese subjects (Sahin-Efe et al., 2018). Elevated levels of irisin in these subjects may be due to irisin resistance developed during the course of obesity (Sahin-Efe et al., 2018). Another study confirmed that obese subjects had significantly increased serum irisin levels compared to normal weight subjects (Pardo et al., 2014). In addition, a 1 kg increment in fat mass could result in a twofold increase in serum irisin levels (Pardo et al., 2014).

Serum irisin levels were positively associated with age, BMI and other metabolic parameters, whereas type 2 diabetic (T2DM) subjects showed significantly decreased serum irisin level irrespective of age or gender (Liu et al., 2013). Similarly, serum irisin levels were significantly decreased in T2DM and negatively associated with newly diagnosed T2DM (Choi et al., 2013). Interestingly, in both *in vivo* and *in vitro* experiments, disassociations resulted depending on the experimental setting (Kurdiova et al., 2014). Serum irisin levels were significantly lower in T2DM, while muscle FNDC5 mRNA expression showed no difference between healthy and diabetic subjects. Meanwhile, FNDC5 mRNA and irisin secretion in the culture supernatant of myotubes from T2DM subjects were significantly higher compared to healthy controls (Kurdiova et al., 2014). The authors assumed that endogenous signals regarding T2DM may play a role in regulating irisin secretion *in vivo* (Kurdiova et al., 2014). In a cross-sectional study including 1115 obese adults, serum irisin was significantly reduced in subjects with metabolic syndrome. Linear regression analysis data revealed that serum irisin levels were negatively associated with fasting insulin, hemoglobin A1c, and albumin/globulin ratios (Yan et al., 2014). Similarly, a study in rats showed that serum irisin was significantly lower in HFD- and streptozotocin-induced diabetic rats compared to healthy rats (Abdelhamid Fathy, 2017). Diabetic rats that underwent chronic exercise training, in this case swimming, exhibited not only increased serum irisin levels and improved blood glucose levels, but also a homeostatic model assessment of insulin resistance compared with sedentary diabetic rats (Abdelhamid Fathy, 2017).

A nested case-controlled study revealed that serum irisin levels were significantly lower in those elderly subjects with obesity, diabetes mellitus and/or hypertension (Guo et al., 2020). Serum irisin showed negative associations with BMI, blood pressure, fasting blood glucose, cholesterol, and triglyceride (Guo et al., 2020). The authors also found that higher levels of serum irisin were associated with reduced risks of hypertension, T2DM, overweight, and obesity (Guo et al., 2020). Animal studies revealed the differential regulatory effects of central and peripheral irisin on blood pressure (Zhang et al., 2015). Intraventricular injection of exogenous irisin increased blood pressure and cardiac contractility via the activation of neurons in the paraventricular nuclei of the hypothalamus, while intravenous injection lowered blood pressure through ATP-sensitive potassium channels in both normal and spontaneous hypertensive rats (Zhang et al., 2015). In congruence with these findings, intravenous injection of irisin lowered the blood pressure of spontaneously hypertensive rats (Fu et al., 2016). Irisin treatment increased nitric oxide production and phosphorylation of endothelial nitric oxide synthase (eNOS) in endothelial cells (Fu et al., 2016). The blood pressure-lowering effect of irisin was found to occur via the AMPK-AKT-eNOS-NO pathway (Fu et al., 2016). These data indicate that irisin plays a protective role in metabolic diseases.

In line with irisin’s role in controlling metabolic diseases, Wagner and colleagues have previously reported the occurrence of obesity phenotypes in mice genetically disrupted for ICAM-1 or αMβ2 (Dong et al., 1997). These findings indicate that some leukocyte integrins and ligands, or their adhesions, are involved in regulating metabolic diseases such as obesity. Thus, one cannot rule out the possibility that there is a functional connection to irisin and the other ligands that may contribute to the regulation of physiological imbalances.

## Conclusion

Integrins on leukocytes and platelets have been validated in controlled clinical trials as therapeutic targets for inflammatory/autoimmune diseases and thrombotic diseases, respectively. In contrast, it has yet to be clinically established whether integrins on tumors and tumor vasculature could serve as therapeutic targets for cancer treatments. A better understanding of how integrins interact with, and signal through, novel ligands present on cancer and cancer stromal cells would help fill the critical knowledge gap currently hampering the development of clinically effective integrin-targeted cancer therapies.

## Author Contributions

EJP and MS contributed to the conceptualization, scope, and outline of this review. EJP, PKM, AI, MGA, SD, EK, and MS analyzed the referenced manuscripts in this manuscript and participated to preparing the manuscript. All authors read and approved the final version.

## Conflict of Interest

The authors declare that the research was conducted in the absence of any commercial or financial relationships that could be construed as a potential conflict of interest.
